# Lipidome of midbody released from neural stem and progenitor cells during mammalian cortical neurogenesis

**DOI:** 10.3389/fncel.2015.00325

**Published:** 2015-08-28

**Authors:** Yoko Arai, Julio L. Sampaio, Michaela Wilsch-Bräuninger, Andreas W. Ettinger, Christiane Haffner, Wieland B. Huttner

**Affiliations:** Max Planck Institute of Molecular Cell Biology and GeneticsDresden, Germany

**Keywords:** neural stem cells, midbody, shotgun lipidomic analysis, phosphatydylserine, mammalian cortical neurogenesis

## Abstract

Midbody release from proliferative neural progenitor cells is tightly associated with the neuronal commitment of neural progenitor cells during the progression of neurogenesis in the mammalian cerebral cortex. While the central portion of the midbody, a cytoplasmic bridge between nascent daughter cells, is engulfed by one of the daughter cell by most cells *in vitro*, it is shown to be released into the extracellular cerebrospinal fluid (CF) *in vivo* in mouse embryos. Several proteins have been involved in midbody release; however, few studies have addressed the participation of the plasma membrane's lipids in this process. Here, we show by Shotgun Lipidomic analysis that phosphatydylserine (PS), among other lipids, is enriched in the released midbodies compared to lipoparticles and cellular membranes, both collected from the CF of the developing mouse embryos. Moreover, the developing mouse embryo neural progenitor cells released two distinct types of midbodies carrying either internalized PS or externalized PS on their membrane. This strongly suggests that phagocytosis and an alternative fate of released midbodies exists. HeLa cells, which are known to mainly engulf the midbody show almost no PS exposure, if any, on the outer leaflet of the midbody membrane. These results point toward that PS exposure might be involved in the selection of recipients of released midbodies, either to be engulfed by daughter cells or phagocytosed by non-daughter cells or another cell type in the developing cerebral cortex.

## Introduction

At the end of mitosis, after the ingression of the cleavage furrow, a dividing cell is partitioned into two daughter cells that remain connected through a cytoplasmic bridge, the midbody, which is composed of the remnants of the central spindle including a set of microtubule interacting proteins (Glotzer, 2009; Chen et al., 2013). Physical separation of daughter cells is achieved by abscission, which completes cell division/cytokinesis by severing the midbody-bridge, bypassing the central portion of the midbody that contains a dense matrix of antiparallel microtubules and electron-dense material (Buck and Tidsale, 1962). Accumulating *in vitro* evidence from induced pluripotent stem and cancer-derived immortalized cells indicates that the post-abscission midbody, with its characteristic matrix composition, is subsequently either retracted asymmetrically by a daughter cell (single abscission) (Kuo et al., 2011) or released into the extracellular space (double abscission). Alternatively, released midbodies could get attached to the surface of a daughter cell and consequently be engulfed (Ettinger et al., 2011; Crowell et al., 2014). In culture cells, neural stem cells, embryonic stem cells, or cancer-derived cells, which are still responsive to differentiation agents, showed midbody-release into the extracellular space (Ettinger et al., 2011). Potentially, these midbodies might be taken up by non-daughter cells, which could result in a long-range dispersion of midbodies. A similar phenomenon is observed in neuroepithelial cells during mouse cortical development (Marzesco et al., 2005; Dubreuil et al., 2007).

At the onset of neurogenesis, neuroepithelial cells divide asymmetrically and preferentially release their midbody (NE midbody in short) into the extracellular ventricular fluid (Marzesco et al., 2005). The NE midbody release showed a strong correlation with the increase of neurogenesis (Dubreuil et al., 2007). Therefore, an efficient release of midbodies from neuroepithelial cells was postulated as a mechanism to reduce proliferative capacity of neuroepithelial cells. Midbody release ultimately results in loss of both the cytoplasmic and membraneous components present in the midbody including lipids from neuroepithelial cells. While the mechanism of the midbody release from neuroepithelial cells had been addressed through protein functions (Dubreuil et al., 2007), it is still unclear what is the fate of the NE midbody during neurogenesis.

In this study, we characterized the NE midbody for the first time through lipidome analysis, where we observed enrichments of specific classes of phosphatidylserine (PS), phosphatidylethanolamine (PE), and specific species of ceramide (Cer), and triacylglycerols (TAGs). Among these, PS was strikingly enriched in NE midbody. It has been proposed that midbodies with PS, particularly carrying externalized PS on their membrane bilayer, are cleaned up by phagocytes in *Caenorhabditis elegans* (Chai et al., 2012). This observation suggests that the PS status on the membrane bilayer is associated with the fate of the NE midbody, which may allow deducing its fate. Our data regarding PS status suggests at least two different categories of recipients, either a daughter cell for an engulfment pathway or non-daughter cells for phagocytosis. The distinct subtype of NE midbodies released upon differentiation (Dubreuil et al., 2007; Ettinger et al., 2011) and carry a stem cell marker (Marzesco et al., 2005; Corbeil et al., 2010) may be a novel cue in and contribute to the complexity of the proliferative territories in the developing cerebral cortex.

## Results

### Midbody enrichment for lipidomic characterization

**Figure 1:**
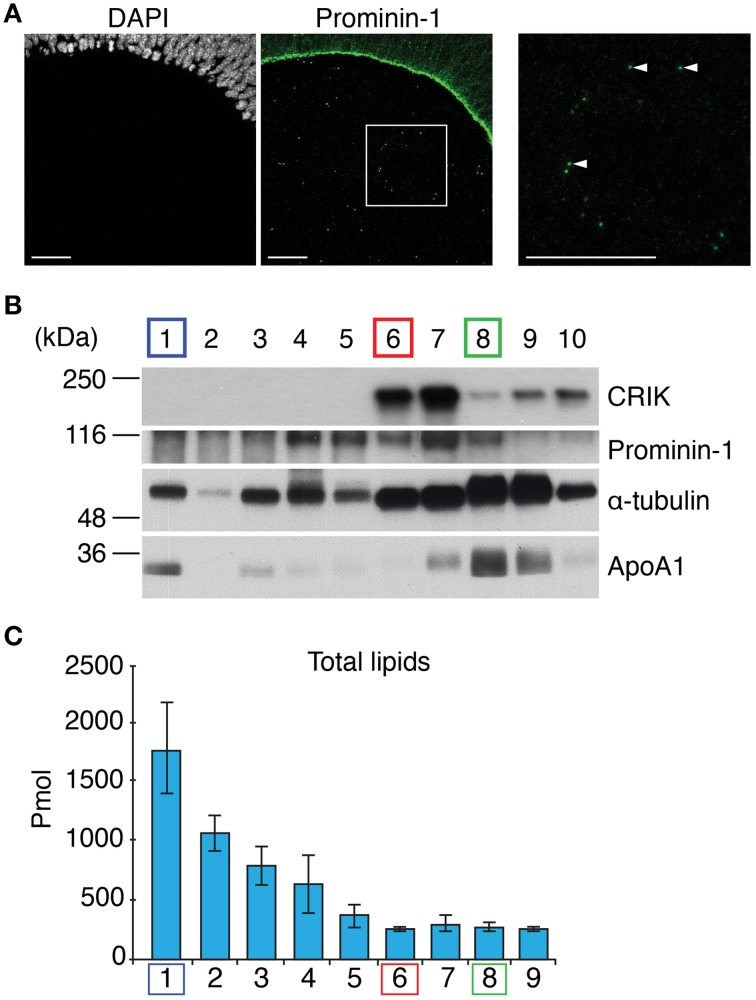
**Midbody enrichment from the E11.5 mouse embryonic cerebrospinal fluid. (A)** Immunofluorescence for prominin-1 (green) combined with DAPI staining (white) of a coronal cryosection (single optical section) of E11.5 dorsal telencephalon. A white box is shown at high magnification in corresponding right panels. Examples of prominin-1-positive midbodies are shown (white arrowheads). Prominin-1-positive midbodies are released into the cerebrospinal fluid. Scale bars, 50 μm. **(B)** Analysis of mouse embryonic cerebrospinal fluid by velocity sucrose density gradient centrifugation and immunoblotting. The lightest density fraction (containing small lipid particles) is indicated as 1 and the densest fraction (containing large materials including likely cell debris and skin tissue fragments) is indicated as 10. Half of each fraction was subjected to immunoblot analysis for CRIK (~197 kDa), Prominin-1 (~115 kDa), α-tubulin (~50 kDa), and Apolipoprotein A1 (~28 kDa). The positions of molecular weight markers are indicated. **(C)** Total lipid quantified from cerebrospinal fluid of E11.5 mouse embryos present in the individual fractions 1–9 after velocity sucrose density gradient.

To enrich midbodies released from neural progenitor cells of the developing mouse embryo (“NE midbody” in short), we collected the ventricular fluid [embryonic cerebrospinal fluid (CF)] of mouse embryos at E11.5 (the number of midbodies is high at this stage) (Marzesco et al., 2005) (Figure 1A). In order to separate the midbody particles (~500 nm) from the biggest lipid “contaminant,” the lipoprotein particles (~50 nm), which are both present in the ventricular fluid (Marzesco et al., 2005), we subjected the sample to a velocity sucrose gradient. We analyzed all fractions from the gradient by immunoblotting for marker proteins prior lipid analysis in order to check for the quality of the separation (Figure 1B). We used the midbody markers: citron rho-interacting kinase (CRIK) (Madaule et al., 1998), prominin-1 and α-tubulin (Dubreuil et al., 2007) in order to identify the fraction enriched in midbody particles in the sucrose fractions. Lipoparticles enriched fractions were identified with the marker apolipoprotein A1 (Furbee et al., 2002) (Figure 1B).

Comparing all fractions in terms of abundance of all markers, we were able to fractionate midbodies (L6) from lipoprotein particles (L1) and cell membranes (L8) (Figure 1B). We identified fraction 6 (L6) as the midbody particle-enriched fraction due to the relative enrichment of midbody markers CRIK, prominin-1 and α-tubulin and the absence of apolipoprotein A1. While fraction 7 (L7) was also enriched with midbody markers, we did not present this fraction as a midbody fraction because of the increased contamination by apolipoprotein A1 as compared to fraction L6, and considered it being a mixed population of L6 and 8. Fraction 1 (L1) was a relatively pure lipoprotein particle-containing fraction judging from the enrichment of α-tubulin and apolipoprotein A1 and depletion of CRIK and prominin-1. Fraction 8 (L8) was used as a reference of a mixed fraction that included low amounts of midbody and plasma membrane of neural progenitor cells due to the presence of all protein markers (Figure 1B). Moreover, we could observe that lipoproteins were by far the most predominant lipid-containing entities present in the ventricular fluid where its purified fraction (L1) outweighs the midbody fraction (L6) by more than seven-fold (Figure 1C).

### Midbodies have a distinct lipid class composition from other cellular membranes

**Figure 2:**
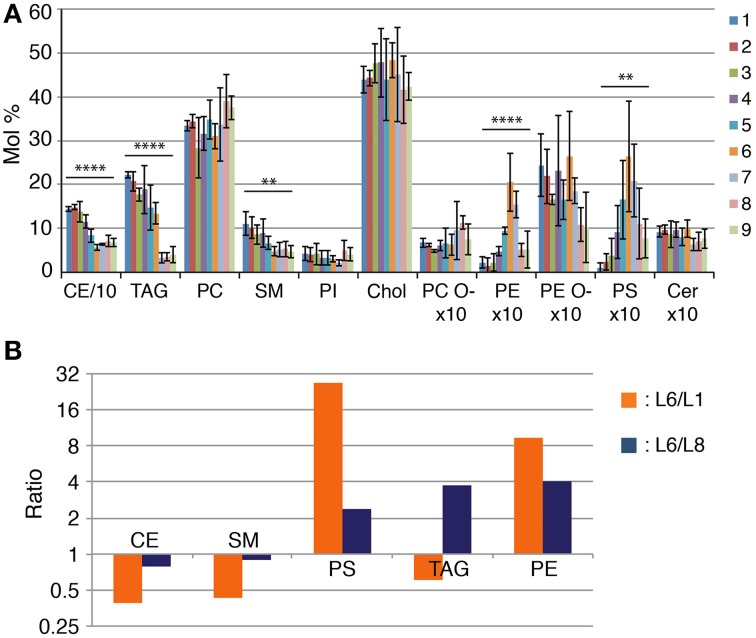
**The Lipidome of NE midbodies. (A)** Lipid class profile of all sucrose fraction. The content of individual lipid classes was determined by summing up absolute abundances of all identified species and is expressed as mol %. We consider 100% to be all the membrane lipids, meaning that CE and TAG are excluded from the normalization. CE and TAG amounts are also normalized to the total membrane lipids. *P*-values were estimated by One-Way ANOVA; ^**^P < 0.01, ^****^P < 0.0001. Error bars correspond to SD (*n* = 3). CE amounts are divided by 10 and PC O-, PE, PE O-, PS, and Cer are multiplied by 10 for visualization purposes. CE, cholesterol ester; TAG, triacylglycerol; PC, phosphatidylcholine; SM, sphingomyelin; PI, phosphatidylinositol; Chol, Cholesterol; PC O- and PE O-, plasmalogens; PE, phosphatidylethanolamine; PS, phosphatidylserine, Cer, ceramide. **(B)** Enrichment of lipid constituency in layer 6 (L6) compared to layer 1 (L1) (L6/L1) and layer 8 (L8) (L6/L8), respectively. The *y*-axis is on a log_2_ scale.

The lipid analysis of the all fractions confirmed that L1 is indeed enriched in lipoprotein particles. Lipoproteins possess a hydrophobic core composed by neutral lipids such as triacylglycerols (TAG) and cholesterol ester (CE) (Willnow et al., 2007) and we observe that this fraction is the most enriched in these lipid classes (Figure 2A). We then see a continuous decrease in the total lipid amount in lower gradient fractions that is accompanied by changes in several lipid classes (Figures 1C, 2A). As expected from fractions more depleted from lipoprotein particles, CEs and TAGs decrease in lower gradient fractions as well as sphingomyelin (SM) (Figure 2A). Most importantly, we observe a sharp increase in phosphatidylethanolamine (PE) and phosphatidylserine (PS) in lower gradient fractions that peak precisely at fraction L6 where we observe the purest midbody fraction (L6) followed by a sharp decrease in the following fractions (Figure 2A). It is important to note that in the fractions below L6, we also observe a dramatic decrease in TAGs and PE plasmalogens (PE O-) and a slight increase in PC (phosphatidylcholine) suggesting high specificity in terms of lipid class composition of the midbody containing fraction compared to the remaining cell membrane fragments. Importantly, the mean PS amount in L6 was 27-fold higher than in L1 (L6, 2.64 ± 1.26; L1, 0.098 ± 0.11, Figure 2B) and 2.4-fold when L6 was compared to the intermediate fraction L8 (L8, 1.11 ± 0.8, Figure 2B). Respective to the amount of PE, the mean PE amount in L6 was 9.3 and four times higher than in L1 and L8, respectively (L6, 2.05 ± 0.66; L1, 0.22 ± 0.13; L8, 0.51 ± 0.15, Figure 2B).

In summary, we observe high specificity of lipid class composition in the midbody-enriched fraction most notably, PS and PE are highly enriched in the midbody-containing fraction compared to that of lipoprotein and cell membrane mixed fractions.

### Specific lipid species are enriched in midbodies compared to other cellular membranes

**Figure 3:**
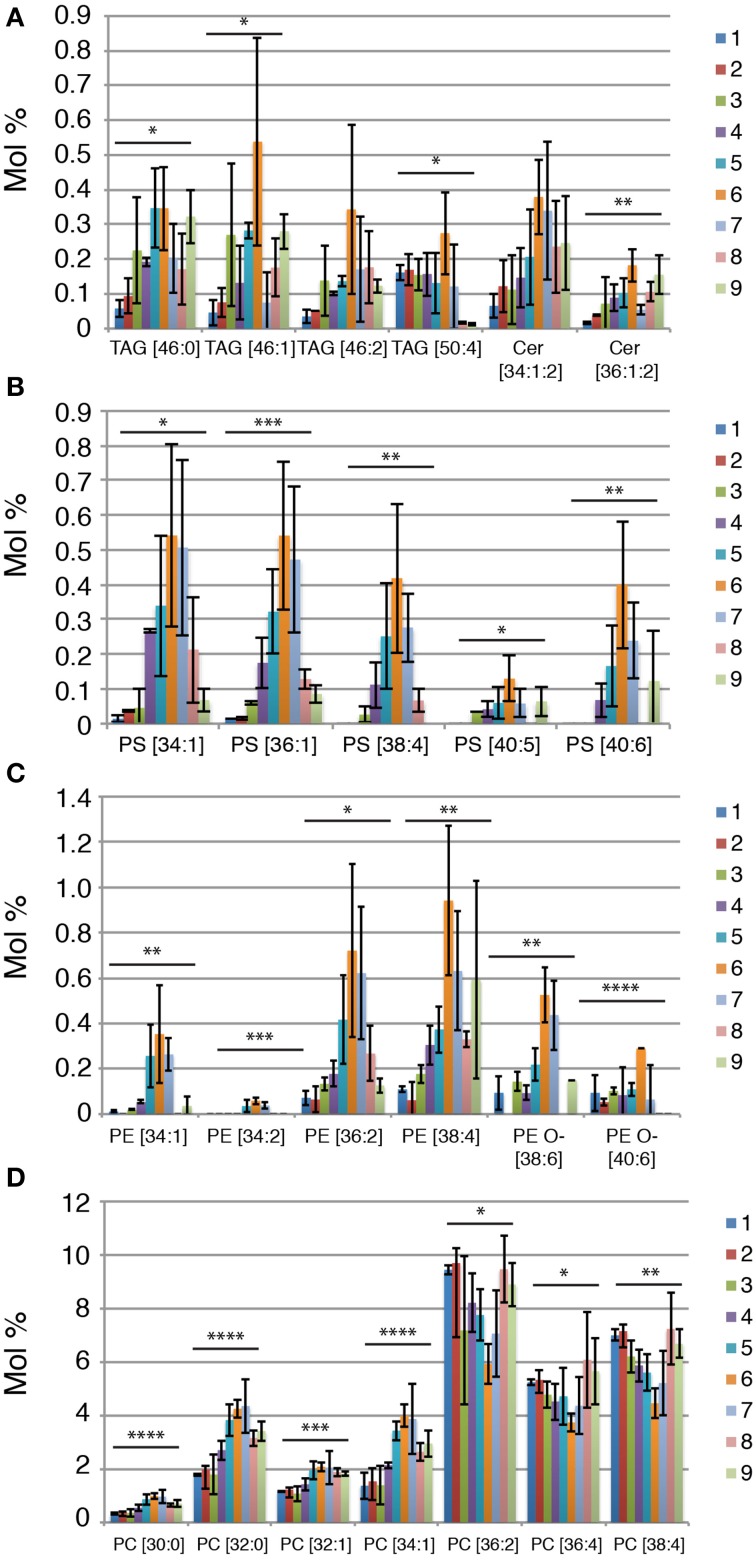
**Lipid species that correlate with the presence of NE midbodies. (A–D)** Lipid species relative abundance (mol %) across the sucrose fractions. Each lipid species is shown as the sum of number of carbon atoms and double bonds present in the long chain base and/or fatty acids. *P*-values were estimated by One-Way ANOVA; ^*^P < 0.05, ^**^P < 0.01, ^***^P < 0.001 and ^****^P < 0.0001. Error bars correspond to SD (*n* = 3).

It has been previously reported that midbodies collected from HeLa cells (“HeLa midbody” in short) showed the specific accumulation of lipid species of C22–24 ceramides, TAG [16:1, 12:0, 18:1] and PS [18:0–20:4] when compared to cells in cytokinesis (Atilla-Gokcumen et al., 2014). In agreement with the result of the HeLa midbody lipidome, the NE midbody lipidome displays an enrichment of TAG [46:2] in the midbody fraction and a specific enrichment of PS [38:4] and ceramides but in our case, we observed instead a specific increase in C16–18 ceramides, Cer [36:1:2] (Figure 3A). Importantly, we could observe many more species enriched in the midbody fraction. Not only we found TAG [46:2] enriched in the midbody fraction, but also many other TAGs with similar short chain fatty acid compositions (C12–18) in different putative combinations, TAG [46:0], TAG [46:1], TAG [46:2], and TAG [50:4] (Figure 3A). We could also observe that most lipid species belonging to PS and PE irrespective of the fatty acid saturation and chain length level display a strong enrichment in the midbody fraction (Figures 3B,C). Interestingly, specific PE O- species, namely with longer and more unsaturated fatty acids, PE O- [38:6] and PE O- [40:6] are enriched in the midbody fraction (Figure 3C). Although PC as a class is not changing significantly between the different fractions, we can observe some changes in lipid species. Shorter and more saturated PC lipid species (C30–34) are enriched in midbodies, PC [30:0], PC [32:0], PC [32:1], and PC [34:1], while longer and more unsaturated lipid species are depleted in the midbody fraction, PC [36:2], PC [36:4], and PC [38:4] (Figure 3D).

In conclusion, we could reproduce the previous published results on lipids enriched in the midbody of Hela cells but identified new midbobody specific lipids, namely within Cer, PS and TAG lipid classes, PE as a lipid class and long and unsaturated PE O- and a remodeling of the PC lipid class to shorter and more saturated lipid species in the midbody.

### NE midbodies carry internalized and externalized PS

From the lipidome analysis, we observed that mainly PC is the most depleted membrane lipid classes (aprox. 8 mol %) while PS and PE are the most enriched lipid classes in NE midbody (almost 5 mol % in total) compared to cell membranes (L8) (Figures 3B–D). If we take into consideration the plasma membrane lipid asymmetry where PC is on the extracellular leaflet of the plasma membrane at the cell surface, and both PS and PE face the cytoplasmic leaflet (Verkleij et al., 1973), it raises the interesting possibility that PS and/or PE might be externalized to keep the membrane integrity. In *C. elegans*, externalization of PS on midbodies released from Q neuroblasts was shown to be an engulfment signal for the midbody via clearance by phagocytes (Chai et al., 2012). These lines of evidence suggest that the status of PS externalization on the midbody might be a mark to infer the fate and/or recipient cell of released midbodies.

**Figure 4:**
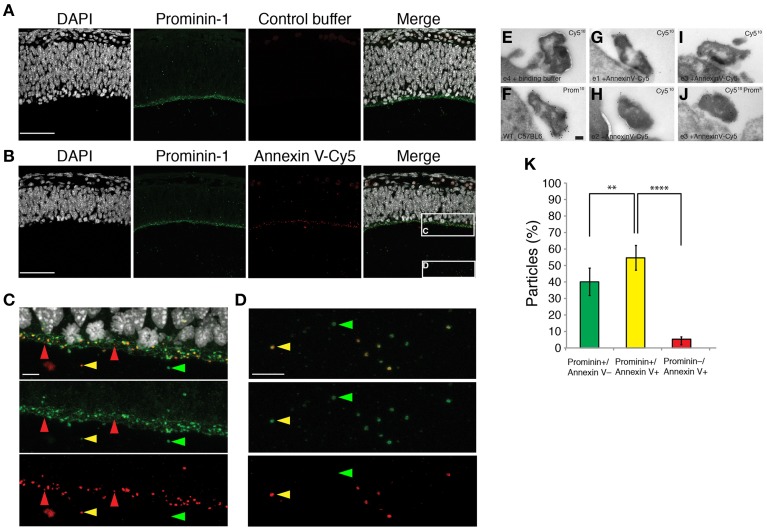
**Phosphatidylserine is on the outer-leaflet of the plasma membrane of the released midbody. (A–D)** Micrographs of mouse embryonic E11.5 coronal cryosections of dorsal telencephalon injected with either Annexin V binding buffer alone **(A)** or containing Annexin V-Cy5 **(B–D)**. Sections were consecutively stained by immunofluorescence for prominin-1 (green) and counterstained with DAPI (white). Images are z-projections of 32 consecutive 0.38-μm optical sections. **(C,D)** Higher magnifications of either the apical plasma membrane of the ventricular zone **(C)** or particles in the cerebrospinal fluid **(D)** [regions indicated by the boxes in **(B)**]. Red arrows, Annexin V-Cy5 single positive particles, green arrows, prominin-1 single positive midbody particles, yellow arrows, Annexin V-Cy5 and prominin-1 double positive midbody particles. Scale bars, 50 μm **(A,B)** and 10 μm **(C,D)**. **(E–J)** Ultrathin cryosections of the apical surface of E11.5 mouse telencephalic neuroepithelium immunogold labeled for Cy-5 **(E,G–I)**, prominin-1 **(F)**, or a combination of Cy-5 and prominin-1 **(J)**. Scale bar, 100 nm. **(K)** Percentage of three different types of particles in the cerebrospinal fluid, classified by decoration with prominin-1 antibody and Annexin V-Cy5. Data are the mean of six 213-μm wide fields from three different embryos and litters; total number of counted particles, 581; error bars indicate SD; ^**^P < 0.01, ^****^P < 0.0001; unpaired *t*-test.

Therefore, we assessed the leaflet localization of PS in midbody membranes, taking advantage of its specific interacting partner, Annexin V (Schutte et al., 1998). To this end, we injected Annexin V directly coupled with Cy5 (Annexin V-Cy5) into the cerebral fluid of mouse embryos at the level of the forming cortex (E11.5), allowing binding for 30 min, followed by fixation and analysis by immunofluorescence. At this early stage of neurogenesis, the fluid filling the ventricular space contains a high amount of released NE midbodies. In this experimental setup, binding of Annexin V-Cy5 to particles is expected only if PS was externalized on the outer leaflet of the membrane bilayer. We confirmed Annexin V-Cy5 positive particles in the ventricular fluid as NE midbodies by a counterstaining with prominin-1, a midbody marker (Marzesco et al., 2005) (Figures 4A–D). In addition to particles in the lumen, we also detected Annexin V-Cy5 staining at the apical plasma membrane of neuroepithelial cells, which is the site of cytokinesis midbody formation between nascent daughter cells (Figure 4C). The presence of Annexin V-Cy5 and prominin-1 double positive midbody structures on the apical surface was confirmed by immunogold labeling and electron microscopy (Figures 4E–J). We found comparably electron-dense, ~500 nm–large structures labeled individually or together by antibodies against prominin-1 or Cy5 (Figures 4E–J). These results indicate that PS externalization could occur at the end of cytokinesis of neuronal progenitor cells.

NE midbodies show three distinct populations defined by their decoration with Annexin V-Cy5 and presence of prominin-1. At the apical surface of the ventricular zone, we observed prominin-1 positive and Annexin V-Cy5 negative particles as a major population (57%, Supplementary Figure 1) and prominin-1 and Annexin V-Cy5 double positive particles as a second population (41%, Supplementary Figure 1). A third minor population (2%) contained only Annexin V-Cy5. Interestingly, in the CF, prominin-1, and Annexin V-Cy5 double positive particles became the major population (55%, Figure 4K) and the prominin-1 positive and Annexin V-Cy5 negative particles were the second population (40%, Figure 4K). The third minor population (5%) contained only Annexin V-Cy5 was still observed. Taken together, these results strongly suggest that a fraction of neural progenitor cell midbodies externalized PS prior to their release in the ventricular fluid and ultimately both PS-externalized and -internalized midbodies were found after cytokinesis.

### PS flipping occurs upon the detachment of the midbody after cytokinesis

In order to determine the dynamics of flipping of PS on the membrane, we analyzed dividing neuroblastoma cells (Neuro-2a), which have been shown to have the capacity of both releasing and engulfing midbodies (Ettinger et al., 2011). We performed live-cell time-lapse microscopy using a transgenic Neuro-2a cell line expressing GFP- tagged mitotic kinesin-like protein-1 (MKLP1) (Poser et al., 2008; Ettinger et al., 2011), a core midbody and central spindle protein (Hu et al., 2012). This cell line allows the visualization of the forming and released midbody (Neuro2a midbody, in short). To visualize externalized PS, we added Annexin V-Cy5 to the culture medium at the beginning of the movie. We assessed PS externalization by appearance of the Cy5 signal upon binding of Annexin V-Cy5. Midbodies were identified as bright spots in the GFP channel.

**Figure 5:**
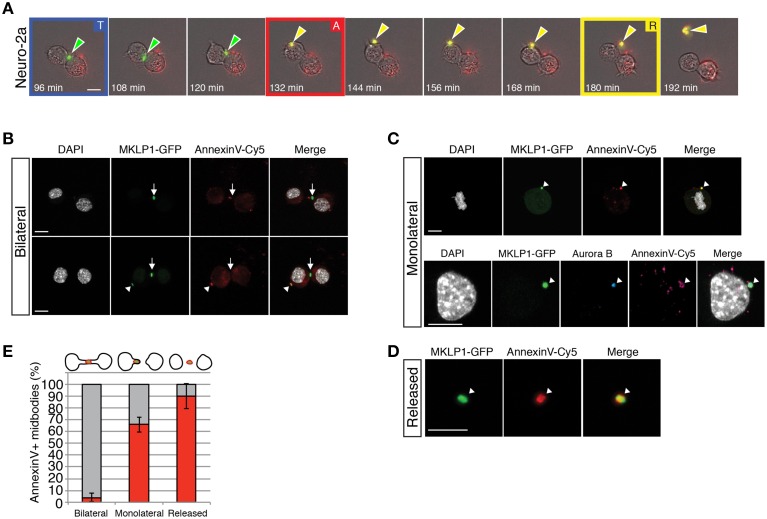
**Phosphatidylserine is maintained on the outer-leaflet of the plasma membrane of retained and released midbodies in MKLP1-GFP Neuro-2a cells. (A)** Individual frames of live-cell time-lapse video microscopy of a MKLP1-GFP stable expressing Neuro-2a cell line recoded at 12 min intervals (Supplementary Movie 1). Selected images of combined bright-field and epifluorescence channels are shown. Green-white arrows show the position of MKLP1-GFP positive midbodies. Yellow-white arrows indicate midbodies that bound to Annexin V-Cy5, which has been added to the medium and interacts with exposed PSs. “T” with blue frame, telophase; “A” with red frame, appearance of a Cy5 spot; “R” with yellow frame, released midbody. Scale bar, 10 μm. **(B–D)** MKLP1-GFP Neuro-2a cells were incubated with AnnexinV-Cy5 for 10 min in culture medium. Images are obtained as z-stacks every 0.38-μm and are maximum intensity projections. Scale bars, 10 μm. **(B**) Examples of bilateral midbodies. Fluorescence for MKLP1-GFP (green) and AnnexinV-Cy5 (red) combined with DAPI staining (white). White arrowheads show Annexin V-Cy5 positive midbodies and white arrows show Annexin V-Cy5 negative midbody. **(C)** Examples of monolateral midbodies. Upper panels, fluorescence for MKLP1-GFP (green), Annexin V-Cy5 (red), and DAPI (white). Lower panels, fluorescence for MKLP1-GFP (green), Aurora B kinase (blue), Annexin V-Cy5 (red), and DAPI (white). White arrowheads, Annexin V-Cy5 positive midbody. **(D)** Example of a released Annexin V-Cy5 positive midbody. Fluorescence for MKLP1-GFP (green) and AnnexinV-Cy5 (red). **(E)** Quantification of MKLP1-GFP and Annexin V-Cy5 colocalization (red columns) vs. non-colocalization (gray columns), expressed as percentage of total number of MKLP1-GFP+ midbodies. Data are the mean four independent experiments, counted in at least 20 randomly chosen fields (total number of counted particles, 243 for bilateral, 234 for monolateral and 137 for released); error bars indicate SD.

As reported previously (Ettinger et al., 2011), MKLP1-GFP localized to the central portion of the midbody in Neuro-2a cells. PS was not externalized during the telophase (Figure 5A, “T”). The midbody was subsequently detached from the central portion of the midbody bridge between daughter cells after either a first or second abscission event of the cytoplasmic membrane. This detachment was identified by the displacement of the midbody from the point of contact of the daughter cell to one of the two daughter cells and PS was externalized at this moment, as determined from appearance of a Cy5 spot (Figure 5A, “A”). Approximately 1 h after the detachment, the midbody was released from a daughter cell (Figure 5A, “R”). These results indicate that PS was flipped out and exposed to the extracellular space at the end of the cytokinesis before midbody release (see Supplementary Movie 1).

We quantified Annexin V and MKLP1-GFP double positive midbodies in Neuro-2a MKLP1-GFP cell cultures in order to understand how widespread this phenomenon is (Figures 5B–E). During late telophase, a midbody bridge is formed between the nascent daughter cells. MKLP1-GFP was recruited to the central portion of the midbody (Figure 5B) as confirmed by immunostaining for α-tubulin to highlight the central spindle microtubules and for Aurora B kinase which localizes to the midzone in anaphase and decorates the inner part of the central spindle in telophase (Supplementary Figure 2A) (Hu et al., 2012). We almost never detected Annexin V-Cy5 staining in these newly formed midbodies (about 3.5% of total bilateral midbodies, Figures 5B,E). Instead, Annexin V was present in midbodies either adhering to one of the daughter cells after a first abscission or on interphase cells (Figures 5B,C, white arrowheads and see Supplementary Figure 2B). The majority of such monolateral midbodies were Annexin V-Cy5 positive (≈66% of total monolateral midbodies, Figure 5E). Fully released Neuro2a midbodies contained Annexin V-Cy5 in the majority of cases (≈90% of total released GFP positive particles, Figures 5D,E). Taken together, PS exposure occurred before the complete release of the midbody and it likely occurred after the first abscission step.

### PS status on HeLa midbodies

**Figure 6:**
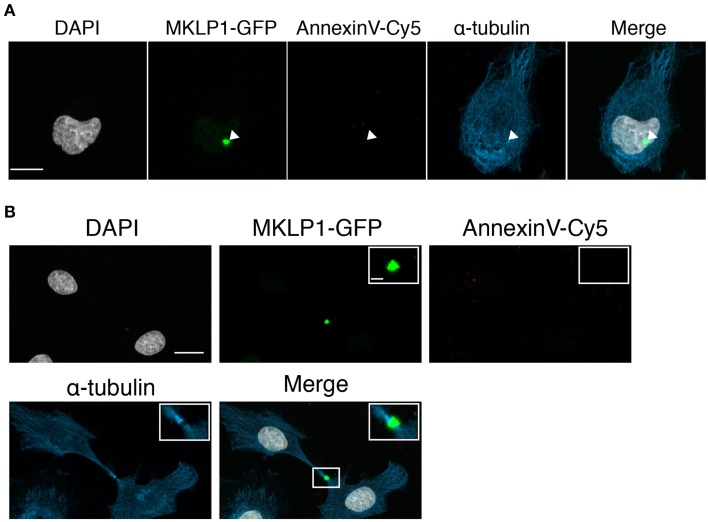
**No AnnexinV-Cy5 colocalization with midbody markers in MKLP1-GFP HeLa cells. (A,B)** MKLP1-GFP HeLa cells were incubated with AnnexinV-Cy5 for 10 min in culture medium. Images were obtained as z-stacks every 0.38-μm and are maximum intensity projections. Shown are individual channels and merge for MKLP1-GFP (green), AnnexinV-Cy5 (red), immunofluorescence for α-tubulin (blue), and DAPI staining (white). Scale bars, 10 μm. **(A)** Example for monolateral midbody. White arrow heads indicate the MKLP1-GFP positive midbody. **(B)** Example for bilateral midbody. Inset, magnification of boxed region in merged channel. Scale bar, 1 μm.

A previous study indicated that half of the total Neuro-2a midbodies are retained on daughter cells and the rest are released into the extracellular space (Ettinger et al., 2011). Conversely, in HeLa cells, midbodies were described to be engulfed by a daughter cell (Crowell et al., 2014). Therefore, we asked whether HeLa midbodies carry externalized PS. Similarly to the analysis of Neuro2a cells, we used a transgenic HeLa cell line expressing MKLP1-GFP to follow the midbody and determined the presence of PS in the outer leaflet of the midbody membrane with Annexin V-Cy5 (Figure 6). Midbody bridges formed during telophase were identified by recruitment of MKLP1-GFP and confirmed by immunostaining for α-tubulin and Aurora B (Supplementary Figure 3). In both monolateral inherited midbodies (Figure 6A) and in newly formed/bilateral midbodies (Figure 6B), we hardly detected PS externalization. These results suggest that PS exposure to the extracellular environment is a characteristic feature of neural-related cell lines and neuronal progenitor cells.

## Discussion

The present study is the first quantitative analysis of the lipid composition of released NE midbodies from mouse embryonic CF. A comparison of lipidome data from HeLa and NE midbodies showed a common enrichment of several lipid species (Atilla-Gokcumen et al., 2014). Among them, the enrichment in specific PS, TAG and Cer species was observed both in NE and HeLa midbodies compared to both lipoprotein particles and dividing cells. In comparison to midbodies from HeLa cells, NE midbodies displayed a wider extent of enrichment of other lipid classes and specific lipid species. This apparent divergence may reflect the functional complexity of the NE midbody in developing mouse embryos, in contrast to cancer-derived, cultured cells like HeLa. It has been shown previously that cultured cells showed a re-arrangement of protein levels involved in metabolic pathways at the expense of other pathways as compared to primary cells, which seems to be required for the adaptation to the cultured conditions (Pan et al., 2009). Particularly relevant for this study is the down-regulation of proteins involved in fatty acid metabolism in cell lines (Pan et al., 2009), a fact that can explain why we identified more varying lipid species in NE midbody when compared to HeLa midbody.

What could be the relevance of the combined enrichment of all the reported lipids for the midbody structure and function? Interestingly, both Ceramide species (C16 and C24-ceramide) identified in our and the previous study (Atilla-Gokcumen et al., 2014), respectively have been shown to contribute for apoptosis (Seumois et al., 2007). Moreover, the enrichment and exposure of PS to the outer leaflet of plasma membrane, a feature observed in midbody membranes, is a common feature of many apoptotic cells (Fadok et al., 1998). These two observations put together suggest that released midbody membranes might contain a potent apoptotic signal and, upon its releasing from the neural stem and progenitor cells and its engulfment by neighboring cells, it might alter the cell fate of both. The strong enrichment in unsaturated PE species in the midbody, is in line with its recognized fusogenic properties and the finding that PE species are exposed on the cell surface specifically at the cleavage furrow during late telophase of cytokinesis (Emoto et al., 1996). PE, due to its unique geometry (inverted cone) which is enhanced by the increase of the degree of unsaturation, in that it forms a non-bilayer structure, is thought to promote rapid phospholipid trans-bilayer movement (Ellens et al., 1989) and stalk membrane formation (Markin et al., 1984) facilitating membrane fusion and fission processes in cellular membranes a process required for midbody release after cytokinesis.

Cells in cytokinesis have been shown to possess more resilient cell membranes requiring three-fold higher forces to provoke their disruption when compared to cells in interphase (Atilla-Gokcumen et al., 2014). This observation is in line with the increase in relative saturation of the most abundant phospholipid in mammalian cells, PC (Figure 2D). We observe enrichment in PC species containing 0 and 1 unsaturations and a decrease in PC species containing two or more unsaturations (Figure 2D). Unsaturation level of lipids is known to alter dramatically the physical properties of cell membranes by changing the membrane fluidity and order [Gennis RB, Biomembranes: Molecular structure and function (Springer Advanced Texts in Chemistry), 1989]. The specific enrichment in short chain TAGs is intriguing. TAGs in general are known to be metabolic energetic reservoirs in cells but the putative different functions of TAGs with specific fatty acid composition are still unknown.

Together, the lipid specificity observed in midbodies, seems to be intimately related to its structure and function. From it, we can conclude that midbodies possess relatively sturdy membranes due to the decrease of saturation of PC species, they are enriched in fusogenic lipids, unsaturated PEs, which facilitate fission and release of midbody, and are also enriched in potent signaling lipids, specific Ceramides and PS species that might influence neighboring cells if engulfed by them. All these observations put together highlight the importance of lipid compositional complexity, specificity and cellular localization in cell biology and how it can be directly associated with structural and functional aspects of cellular processes (Simons and Sampaio, 2011).

Although differences in lipid composition exist, common lipid species suggest shared characteristics between NE and HeLa midbodies. In HeLa cells, midbodies are known to be engulfed by a daughter cell through an actin-dependent phagocytosis-like mechanism (Crowell et al., 2014) while the release of midbody from neural-derived cells (Ettinger et al., 2011) seems not compatible with the engulfment by one of the daughter cells. PS is a structural phospholipid, which exists in the cytoplasmic leaflet of the plasma membrane bilayer and whose externalization has been associated with phagocytosis during apoptotic body clearance (Fadok et al., 1998), retinal rod and cone photoreceptors clearance (Kevany and Palczewski, 2010) and also in midbody clearance in *C. elegans* (Chai et al., 2012). In above examples, the phagocytosis of PS-externalized midbodies was carried out by non-daughter cells. Hence, it is possible that PS-externalized NE midbodies may be engulfed by phagocytes in the developing mouse embryo. It has been proposed, however, that other cell types in the developing mouse central nervous system such as microglia, astrocytes and even neural progenitor cells or neurons have the potential to behave as phagocytes due to the expression of phagocytic receptors (Sokolowski and Mandell, 2011). One of the phagocytic receptors, Gas6, which binds to PS, is indeed expressed highly in proliferative neural progenitor cells compared to neurogenic progenitor cells (Arai and Huttner, unpublished data). These lines of evidence support the idea that PS-externalized NE midbodies could be phagocytosed by sister neural progenitor cells through a phagocytosis-related mechanism using Gasp6 signaling. We previously reported that midbodies carrying the stem cell marker prominin-1 (Marzesco et al., 2005) were released from proliferative neural progenitor cells, which may contribute to their loss of stemness (Dubreuil et al., 2007). One possibility is that PS-externalized midbody could be engulfed by proliferative neural progenitor cells to maintain a balance for the stem cell capacity or serving as a means of communicating between the proliferative territories of the developing mouse brain. In addition to the PS externalized NE midbody, we also observed PS internalized NE midbodies (Figure 4K), which may reflect the heterogeneity of neural progenitor cells providing these two different types of midbodies. With the progression of neurogenesis, proliferative neural progenitor cells give rise to neuronally committed neurogenic progenitor cells (Haubensak et al., 2004). It would be interesting to identify which type of neural progenitor cells preferentially release either PS-externalized or -internalyzed midbodies.

What could be the fate of the biological components of NE midbodies after cells take them up? The engulfed midbody should be digested in lysosomes into monomolecular species, which are recycled if necessary. Fatty acids from the PS species of the NE midbody could also be recycled, if any, it participates on any metabolic pathways. Amongst the PS species identified in our lipidomics, the presence of omega-3 and 6 fatty acids that cannot be produced *de novo* have been strongly suggested, for instance, (PS [38:4] = PS [18:0–20:4], arachidonic acid [20:4]) (Sampaio et al., 2011). Therefore, the recycling of midbodies could participate to the essential lipid metabolism during cortical development, participating to the maintenance of a lipid species pool for controlling proper neurogenesis.

## Methods

### Animals

All mouse embryos used were wildtype C57BL/six mice obtained by overnight mating of wildtype C57BL/six males and females. Noon of the day on which the vaginal plug was observed was defined as embryonic day (E) 0.5. All animal studies were conducted in accordance with German animal welfare legislation, and the necessary licenses obtained from the regional Ethical Commission for Animal Experimentation of Dresden, Germany.

### Enrichment of the midbody fraction using velocity sucrose density gradient centrifugation

E11.5 C57BL/6 mouse embryos were dissected in ice-cold 150 mM NH_4_Ac dissolved in lipid mass spectrometry-grade water (Merck; LiChrosolv grade). CF was collected as described (Marzesco et al., 2005). 1–2 μl of CF per embryo were collected and pooled in a 1.5 ml Eppendorf tube (Eppendorf) on ice. Seventy-six micro liter of CF were mixed with 4 μl of 100x proteinase inhibitor mix (Roche) and 320 μl of 2.5 M sucrose in water to yield a total of 400 μl of CF mixture at a final concentration of 2 M sucrose. The CF mix was transferred into the bottom of an ultraclean ultracentrifugation tube (Beckman coulter). The gradient was prepared by adding layers of less dense sucrose solutions (400 μl of 1.6 M, 400 μl of 1 M, 300 μl of 0.3 M, and 300 μl of 0.1 M) upon one another. The samples were centrifuged in a TLS55 swinging rotor (Beckman coulter) at 50,000 rpm for 18 h at 4°C with a no deceleration profile. Immediately after the centrifugation, 180 μl fractions were collected into 1.5 ml Eppendorf tubes on ice. Ten micro liter of each fraction were used to determine the sucrose concentration in the fraction, 85 μl were used each for Western blotting and lipid mass spectrometry. In case of electron microscopy (EM) analysis, 10 μl of each fraction were fixed overnight at 4°C in 0.5% of EM grade glutaraldehyde/4% paraformaldehyde in 0.12 M sodium phosphate buffer, pH 7.2.

### Protein and lipid precipitations from sucrose samples

For immunoblotting, samples were subjected to methanol-chloroform precipitation by mixing samples in 1:4:1:3 ratio (sample:methanol:chloroform:water). Protein was precipitated by gently vortexing the tube followed by centrifugation at 13,000 rpm for 5 min at room temperature (r.t.). The upper layer was removed without touching the interface layer. Two-hundred and fifty-five liter of methanol were added (3 × volume of the original sample volume, 85 μl). Proteins were pelleted by centrifugation at 13,000 rpm for 5 min at r.t. and resuspended in 2x concentrated SDS Laemmli sample buffer. Protein samples were denatured by boiling at 95°C for 5 min and stored at −20°C.

For lipid extraction, 85 μl sample from each fraction were transferred into a 1.5 ml Eppendorf tube filled with 157 mM of ammonium bicarbonate (NH_4_HCO_3_) up to 1.5 ml. Particles were pelleted in the TLA55 fixed-angle rotor (Beckman coulter) at 30,000 rpm for 4 h at 4°C. Removed NH_4_HCO_3_ and pelleted lipid samples were frozen in liquid nitrogen and stored at −80°C.

### Lipid extraction for lipid mass spectrometry

Lipid samples were mixed with 200 μl of 157 mM of NH4HCO3 and vortexed at 1400 rpm at 4°C. Ten micro liter of an internal standard lipid mix (Avanti Polar lipids; PC, PE, PS, PG, PA, Cer, SM, GalCer, LacCer, Chol, and DAG) was added to each sample. To extract all lipids, 1 ml of a 2.75:1 mixture of chloroform/methanol was added to each tube and vortexed at 1400 rpm for 1 h at 4°C. Fractions with high sucrose content (>1 M) were “pre-washed” with 200 μl of MS grade water before the extraction to remove excess sucrose. After extraction, sample tubes were centrifuged at 1000 g for 2 min at 4°C. Bottom phases were transferred into fresh 1.5 ml Eppendorf tubes (10:1 lipid fraction). The organic solvent was evaporated using a vacuum centrifuge. Dried samples were re-suspended with 100 μl of a 1:2 mixture of chloroform/methanol and vortexed for 10 min at 4°C. This re-suspension solution was mixed with MS compatible polarity specific buffers to optimize for ionization of specific lipids as described previously (Carvalho, 2012). The data was acquired on a LTQ-Orbitrap using high mass resolution *R*_*m*−*z* = 400_ = 100, 000 and the spectra was analyzed with LipidXplorer software (Herzog et al., 2011, 2012).

### Immunofluorescence, electron microscopy, annexin V-Cy5 injection, and image acquisition

Living embryos at E11.5 were dissected out of the uterus with the placenta intact and transferred into 1x Tyrode solution (Sigma-Aldrich). AnnexinV-Cy5 solution (BioVision) was diluted to 1:300 in 1x binding buffer (10 mM Hepes-NaOH pH7.4 containing 140 mM NaCl and 2.5 mM CaCl_2_) and injected into telencephalic vesicles of living embryos (1x concentrated binding buffer was used as control). Injected embryos were incubated at r.t. for 30 min (room air and protected from light). Whole embryos were fixed for 20–25 h at 4°C in 4% paraformaldehyde in 0.12 M sodium phosphate buffer, pH 7.2. For cryosectioning, embryos were equilibrated for 12–24 h at 4°C in 30% sucrose in PBS, embedded in Tissue-Tek O.C.T compound (Sakura Finetek), and stored at −20°C. Cryostat sections (10 μm) were rehydrated with PBS, quenched with 50 mM NH_4_CL for 30 min, washed with 0.01% Digitonin (Sigma-Aldrich) in PBS for 30 min and blocked with 3% BSA in 0.01% Digitonin/PBS for 30 min at r.t. Sections were washed with 1% BSA in 0.01% Digitonin/PBS and incubated overnight at 4°C with first primary antibody, followed by incubation for 1 h at r.t. with fluorescently labeled secondary antibodies and DAPI (4′,6-diamidino-2-phenylindole, Roche) in 1% BSA in 0.01% Digitonin/PBS. Sections were mounted in Mowiol. For electron microscopy, E11.5 dorsal telencephalon was analyzed by transmission EM, with pre- or post-embedding prominin-1 immunolabeling.

The following primary antibodies were used: rabbit anti-Prominin-1 [αD, MPI-CBG; 1:20,000 for immunoblotting (IB)]; goat antibody against apolipoprotein A1 (Biodesign; 1:1000); mouse monoclonal antibodies against CRIK [BD bioscience; 1:200 for immunofluorescence (IF) and 1:4000 for IB], α-tubulin (Sigma; 1:300 for IF and 1:60,000 for IB) and Aurora B (BD bioscience; 1:300) and rat anti-Prominin-1 (13A4, MPI-CBG; 1:300 for IF). Secondary antibodies were: horseradish peroxidase-coupled antibodies (Jackson laboratory), Alexa Fluor 488, 555, and 647 labeled IgG antibodies (Invitrogen).

Fluorescence images were acquired by confocal microscopy (Zeiss LSM 700) and images were acquired with a Zeiss Plan-Apochromat 20x, 0.8 NA and a Zeiss C-Apochromat 40x, 1.2 NA objectives.

### Live-cell time-lapse microscopy

The protocol for this time-lapse microscopy has been described previously (Ettinger et al., 2011). Briefly, Neuro-2a MKLP1-GFP cells were grown on chambered coverslips (Lab-Tek, Nunc) in DMEM-GlutaMax medium supplemented with 10% FCS and incubated for 24 h at 37°C with 5% CO_2_. The medium was replaced with Annexin V-Cy5 containing medium (1:5000) and cells were imaged with a Zeiss Axiovert 200 M inverted epifluorescence microscope equipped with a motorized stage, incubator, humidifier, and carbon dioxide supply (Visitron Systems, Germany). Bright-field and fluorescence images were acquired using a C-Apochromat 40x water immersion objective (NA 1.4) and recorded with a Roper Scientific Cool SNAP ES CCD camera using MetaMorph software. Multiple fields were acquired in parallel per experiment at 12 min intervals for up to 24 h.

### Annexin V-Cy5 incubation with the Neuro-2a-MKLP1 transgenic cell line

Neuro-2a MKLP1-GFP cells were grown on poly-L-lysine coated coverslips in DMEM-GlutaMax medium supplemented with 10% FCS and incubated for 24 h at 37°C with 5% CO_2_. The medium was replaced with Annexin V-Cy5 containing medium (1:2500), incubated for 10 min at r.t. protected from light, and fixed with 4% paraformaldehyde in 0.12 M sodium phosphate buffer, pH 7.2. Cells were further processed for immunofluorescence like sections of mouse embryos.

## Author contributions

YA designed and performed the experiments, analyzed the data and co-wrote the manuscript, JS designed and performed lipid mass spectrometry, analyzed the data and co-wrote the manuscript, MB performed EM and co-wrote the manuscript, AE helped and performed time-lapse imaging, and co-wrote the manuscript, CH performed experiments and WH analyzed data, co-wrote the manuscript and supervised project. The authors declare that they have no competing financial interests.

### Conflict of interest statement

The authors declare that the research was conducted in the absence of any commercial or financial relationships that could be construed as a potential conflict of interest.

## Supplementary material

Supplementary Figure 1 **Phosphatidylserine is on the outer-leaflet of the apical plasma membrane of the neuronal progenitor cells. (A,B)** Micrographs of mouse embryonic E11.5 coronal cryosections of dorsal telencephalon injected with Annexin V-Cy5 binding buffer. Sections were consecutively stained by immunofluorescence for prominin-1 (green) and counterstained with DAPI (white). Images are z-projections of 32 consecutive 0.38-μm optical sections. **(B)** Higher magnifications of the apical plasma membrane of the ventricular zone [boxed region indicated in **(A)**]. Red-white arrows, Annexin V-Cy5 single positive particles, green arrows, prominin-1 single positive midbody particles, yellow arrows, Annexin V-Cy5 and prominin-1 double positive midbody particles. Scale bars, 50 μm **(A)** and 10 μm **(B)**. **(C)** Percentage of three different types of particles on the apical plasma membrane of the ventricular zone, classified by decoration with prominin-1 positive and Annexin V-Cy5 positive signal. Data are the mean of six 213-μm wide fields from three different embryos and litters; total number of counted particles, 455 for prominin+ AnnexinV−, 354 for prominin+ AnnexinV+ and 16 for prominin− AnnexinV+; error bars indicate SD; ^*^*P* < 0.05, ^****^*P* < 0.0001; unpaired *t*-test.

Supplementary Figure 2 **Expression of midbody markes in MKLP1-GFP Neuro-2a cells. (A,B)** Fluorescence for MKLP1-GFP (green), immunofluorescence for α-tubulin and Aurora B kinase (red) in Neuro-2a MKLP1-GFP cells during telophase **(A)** and interphase **(B)** combined with DAPI staining (white). Images are maximum intensity projections of z-stacks of optical sections taken every 0.38 μm. Insets, magnifications of bilateral **(A)** and monolateral **(B)** midbodies. Scale bars, 10 μm and 1 μm (inset).

Supplementary Figure 3 **Expression of midbody markers in MKLP1-GFP HeLa cells. (A)** Fluorescence for MKLP1-GFP (green) and immunofluorescence for α-tubulin in telophase (2 upper panels) and interphase (lower panels) cells combined with DAPI staining (white). Scale bars, 10 μm. **(B)** Fluorescence for MKLP1-GFP (green) and immunofluorescence for Aurora B kinase in telophase (upper panels) and interphase (lower panels) cells having midbody combined with DAPI staining (white). Scale bars, 10 μm. Insets, magnifications of newly formed midbodies with telophase-bridge between the two daughter cells intact [upper panels in **(A,B)**] or inherited, monolateral midbodies with no apparent cytoplasmic bridge to the other daughter cell [lower panels in **(A,B)**]. Scale bars, 1 μm.

## References

[B1] Atilla-Gokcumen G. E. Muro E. Relat-Goberna J. Sasse S. Bedigian A. Coughlin M. L.. (2014). Dividing cells regulate their lipid composition and localization. Cell 156, 428–439. 10.1016/j.cell.2013.12.015 24462247 PMC3909459

[B2] Buck R. C. Tidsale J. M. (1962). An electron microscopic study of the cleavage furrow in mammalian cells. J. Cell Biol. 13, 117–125. 10.1083/jcb.13.1.117 13874302 PMC2106067

[B3] Carvalho V. M. (2012). The coming of age of liquid chromatography coupled to tandem mass spectrometry in the endocrinology laboratory. J. Chromatogr. B Analyt. Technol. Biomed. Life. Sci. 883–884, 50–58. 10.1016/j.jchromb.2011.08.027 21907642

[B4] Chai Y. Tian D. Yang Y. Feng G. Cheng Z. Li W.. (2012). Apoptotic regulators promote cytokinetic midbody degradation in *C*. elegans. J. Cell Biol. 199, 1047–1055. 10.1083/jcb.201209050 23253479 PMC3529525

[B5] Chen C.-T. Ettinger A. W. Huttner W. B. Doxsey S. J. (2013). Resurrecting remnants: the lives of post-mitotic midbodies. Trends Cell Biol. 23, 118–128. 10.1016/j.tcb.2012.10.012 23245592 PMC4196272

[B6] Corbeil D. Marzesco A.-M. Wilsch-Bräuninger M. Huttner W. B. (2010). The intriguing links between prominin-1 (CD133), cholesterol-based membrane microdomains, remodeling of apical plasma membrane protrusions, extracellular membrane particles, and (neuro)epithelial cell differentiation. FEBS Lett. 584, 1659–1664. 10.1016/j.febslet.2010.01.050 20122930

[B7] Crowell E. F. Gaffuri A.-L. Gayraud-Morel B. Tajbakhsh S. Echard A. (2014). Engulfment of the midbody remnant after cytokinesis in mammalian cells. J. Cell Sci. 127, 3840–3851. 10.1242/jcs.154732 25002399

[B8] Dubreuil V. Marzesco A.-M. Corbeil D. Huttner W. B. Wilsch-Bräuninger M. (2007). Midbody and primary cilium of neural progenitors release extracellular membrane particles enriched in the stem cell marker prominin-1. J. Cell Biol. 176, 483–495. 10.1083/jcb.200608137 17283184 PMC2063983

[B9] Ellens H. Siegel D. P. Alford D. Yeagle P. L. Boni L. Lis L. J.. (1989). Membrane fusion and inverted phases. Biochemistry 28, 3692–3703. 10.1021/bi00435a011 2751990

[B10] Emoto K. Kobayashi T. Yamaji A. Aizawa H. Yahara I. Inoue K.. (1996). Redistribution of phosphatidylethanolamine at the cleavage furrow of dividing cells during cytokinesis. Proc. Natl. Acad. Sci. U.S.A. 93, 12867–12872. 10.1073/pnas.93.23.12867 8917511 PMC24012

[B11] Ettinger A. W. Wilsch-Bräuninger M. Marzesco A.-M. Bickle M. Lohmann A. Maliga Z.. (2011). Proliferating versus differentiating stem and cancer cells exhibit distinct midbody-release behaviour. Nat. Commun. 2, 503. 10.1038/ncomms1511 22009035 PMC3207209

[B12] Fadok V. A. Bratton D. L. Frasch S. C. Warner M. L. Henson P. M. (1998). The role of phosphatidylserine in recognition of apoptotic cells by phagocytes. Cell Death Differ. 5, 551–562. 10.1038/sj.cdd.4400404 10200509

[B13] Furbee J. W. Francone O. Parks J. S. (2002). *In vivo* contribution of LCAT to apolipoprotein B lipoprotein cholesteryl esters in LDL receptor and apolipoprotein E knockout mice. J. Lipid Res. 43, 428–437. 11893779

[B14] Glotzer M. (2009). The 3Ms of central spindle assembly: microtubules, motors and MAPs. Nat. Rev. Mol. Cell Biol. 10, 9–20. 10.1038/nrm2609 19197328 PMC2789570

[B15] Haubensak W. Attardo A. Denk W. Huttner W. B. (2004). Neurons arise in the basal neuroepithelium of the early mammalian telencephalon: a major site of neurogenesis. Proc. Natl. Acad. Sci. U.S.A. 101, 3196–3201. 10.1073/pnas.0308600100 14963232 PMC365766

[B16] Herzog R. Schuhmann K. Schwudke D. Sampaio J. L. Bornstein S. R. Schroeder M.. (2012). LipidXplorer: a software for consensual cross-platform lipidomics. PLoS ONE 7:e29851. 10.1371/journal.pone.0029851 22272252 PMC3260173

[B17] Herzog R. Schwudke D. Schuhmann K. Sampaio J. L. Bornstein S. R. Schroeder M.. (2011). A novel informatics concept for high-throughput shotgun lipidomics based on the molecular fragmentation query language. Genome Biol. 12:R8. 10.1186/gb-2011-12-1-r8 21247462 PMC3091306

[B18] Hu C.-K. Coughlin M. Mitchison T. J. (2012). Midbody assembly and its regulation during cytokinesis. Mol. Biol. Cell 23, 1024–1034. 10.1091/mbc.E11-08-0721 22278743 PMC3302730

[B19] Kevany B. M. Palczewski K. (2010). Phagocytosis of retinal rod and cone photoreceptors. Physiology (Bethesda) 25, 8–15. 10.1152/physiol.00038.2009 20134024 PMC2839896

[B20] Kuo T.-C. Chen C.-T. Baron D. Onder T. T. Loewer S. Almeida S.. (2011). Midbody accumulation through evasion of autophagy contributes to cellular reprogramming and tumorigenicity. Nat. Cell Biol. 13, 1214–1223. 10.1038/ncb2332 21909099 PMC4208311

[B21] Madaule P. Eda M. Watanabe N. Fujisawa K. Matsuoka T. Bito H.. (1998). Role of citron kinase as a target of the small GTPase Rho in cytokinesis. Nature 394, 491–494. 10.1038/28873 9697773

[B22] Markin V. S. Kozlov M. M. Borovjagin V. L. (1984). On the theory of membrane fusion. The stalk mechanism. Gen. Physiol. Biophys. 3, 361–377. 6510702

[B23] Marzesco A.-M. Janich P. Wilsch-Bräuninger M. Dubreuil V. Langenfeld K. Corbeil D.. (2005). Release of extracellular membrane particles carrying the stem cell marker prominin-1 (CD133) from neural progenitors and other epithelial cells. J. Cell Sci. 118, 2849–2858. 10.1242/jcs.02439 15976444

[B24] Pan C. Kumar C. Bohl S. Klingmueller U. Mann M. (2009). Comparative proteomic phenotyping of cell lines and primary cells to assess preservation of cell type-specific functions. Mol. Cell. Proteomics 8, 443–450. 10.1074/mcp.M800258-MCP200 18952599 PMC2649808

[B25] Poser I. Sarov M. Hutchins J. R. A. Hériché J.-K. Toyoda Y. Pozniakovsky A.. (2008). BAC TransgeneOmics: a high-throughput method for exploration of protein function in mammals. Nat. Methods 5, 409–415. 10.1038/nmeth.1199 18391959 PMC2871289

[B26] Sampaio J. L. Gerl M. J. Klose C. Ejsing C. S. Beug H. Simons K.. (2011). Membrane lipidome of an epithelial cell line. Proc. Natl. Acad. Sci. U.S.A. 108, 1903–1907. 10.1073/pnas.1019267108 21245337 PMC3033259

[B27] Schutte B. Nuydens R. Geerts H. Ramaekers F. (1998). Annexin V binding assay as a tool to measure apoptosis in differentiated neuronal cells. J. Neurosci. Methods 86, 63–69. 10.1016/S0165-0270(98)00147-2 9894786

[B28] Seumois G. Fillet M. Gillet L. Faccinetto C. Desmet C. François C.. (2007). *De novo* C16- and C24-ceramide generation contributes to spontaneous neutrophil apoptosis. J. Leukoc. Biol. 81, 1477–1486. 10.1189/jlb.0806529 17329567

[B29] Simons K. Sampaio J. L. (2011). Membrane organization and lipid rafts. Cold Spring Harb. Perspect. Biol. 3:a004697. 10.1101/cshperspect.a004697 21628426 PMC3179338

[B30] Sokolowski J. D. Mandell J. W. (2011). Phagocytic clearance in neurodegeneration. Am. J. Pathol. 178, 1416–1428. 10.1016/j.ajpath.2010.12.051 21435432 PMC3078427

[B31] Verkleij A. J. Zwaal R. F. Roelofsen B. Comfurius P. Kastelijn D. van Deenen L. L. (1973). The asymmetric distribution of phospholipids in the human red cell membrane. A combined study using phospholipases and freeze-etch electron microscopy. Biochim. Biophys. Acta 323, 178–193. 10.1016/0005-2736(73)90143-0 4356540

[B32] Willnow T. E. Hammes A. Eaton S. (2007). Lipoproteins and their receptors in embryonic development: more than cholesterol clearance. Developement 134, 3239–3249. 10.1242/dev.004408 17720693

