# Supplementary figures and images for: Lipidome of midbody released from neural stem and progenitor cells during mammalian cortical neurogenesis
Source: Front Cell Neurosci. 2015 Aug 28;9:325. doi: 10.3389/fncel.2015.00325 (PMC4551859; doi:10.3389/fncel.2015.00325)

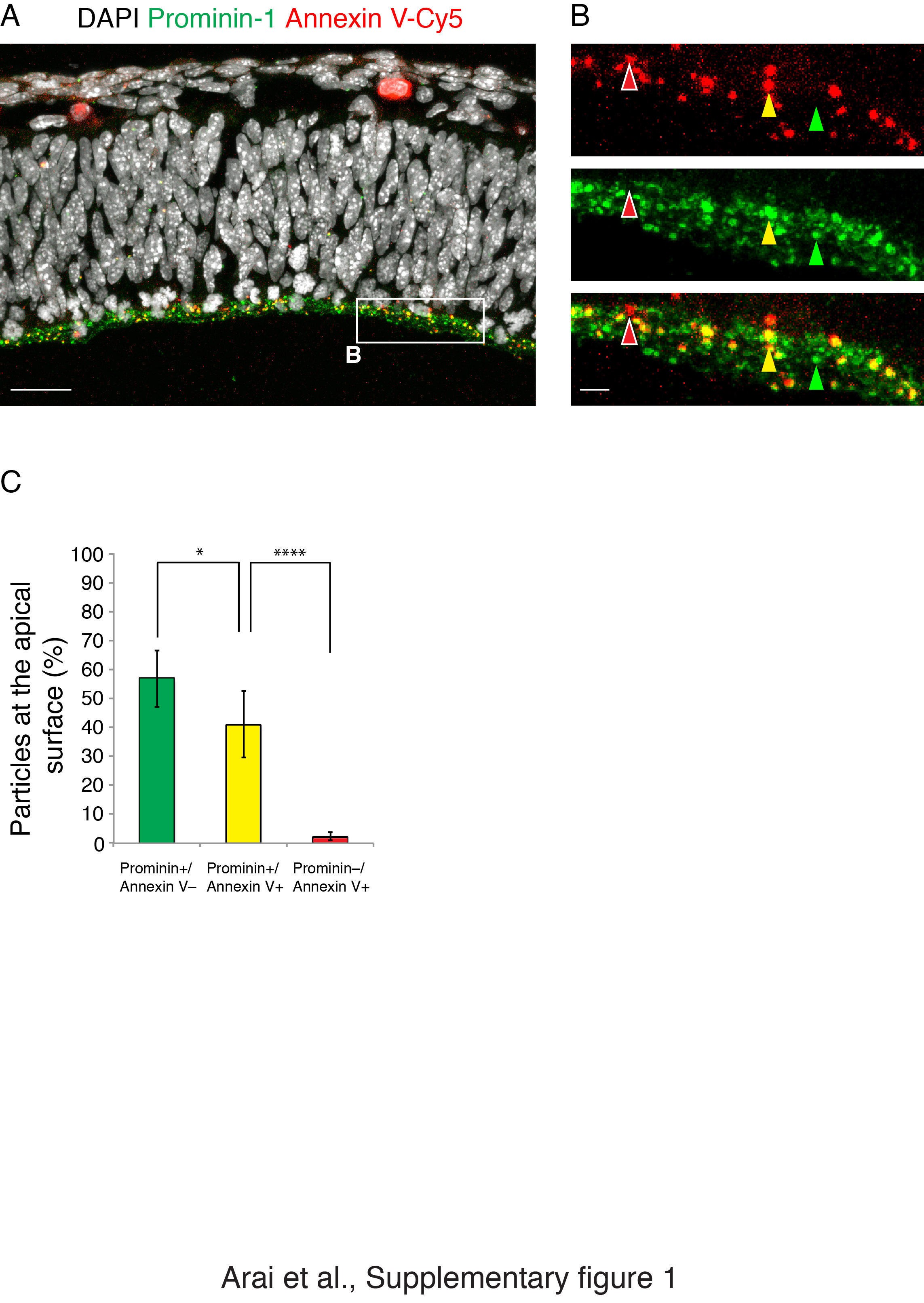

Supplement: Supplementary Figure 1 — Phosphatidylserine is on the outer-leaflet of the apical plasma membrane of the neuronal progenitor cells. (A,B) Micrographs of mouse embryonic E11.5 coronal cryosections of dorsal telencephalon injected with Annexin V-Cy5 binding buffer. Sections were consecutively stained by immunofluorescence for prominin-1 (green) and counterstained with DAPI (white). Images are z-projections of 32 consecutive 0.38-μm optical sections. (B) Higher magnifications of the apical plasma membrane of the ventricular zone [boxed region indicated in (A)]. Red-white arrows, Annexin V-Cy5 single positive particles, green arrows, prominin-1 single positive midbody particles, yellow arrows, Annexin V-Cy5 and prominin-1 double positive midbody particles. Scale bars, 50 μm (A) and 10 μm (B). (C) Percentage of three different types of particles on the apical plasma membrane of the ventricular zone, classified by decoration with prominin-1 positive and Annexin V-Cy5 positive signal. Data are the mean of six 213-μm wide fields from three different embryos and litters; total number of counted particles, 455 for prominin+ AnnexinV−, 354 for prominin+ AnnexinV+ and 16 for prominin− AnnexinV+; error bars indicate SD; *P < 0.05, ****P < 0.0001; unpaired t-test. [file Image1.JPEG]

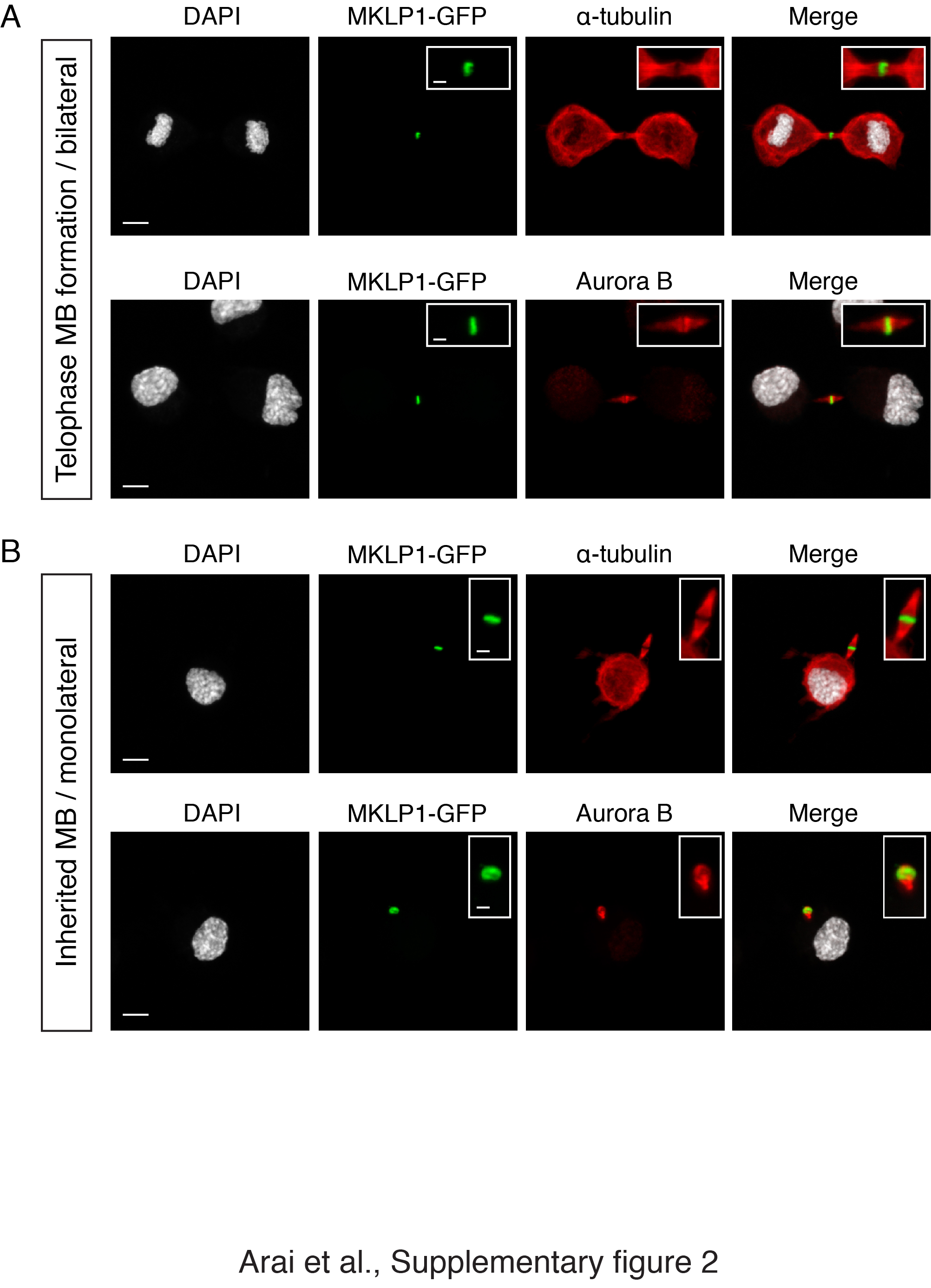

Supplement: Supplementary Figure 2 — Expression of midbody markes in MKLP1-GFP Neuro-2a cells. (A,B) Fluorescence for MKLP1-GFP (green), immunofluorescence for α-tubulin and Aurora B kinase (red) in Neuro-2a MKLP1-GFP cells during telophase (A) and interphase (B) combined with DAPI staining (white). Images are maximum intensity projections of z-stacks of optical sections taken every 0.38 μm. Insets, magnifications of bilateral (A) and monolateral (B) midbodies. Scale bars, 10 μm and 1 μm (inset). [file Image2.JPEG]

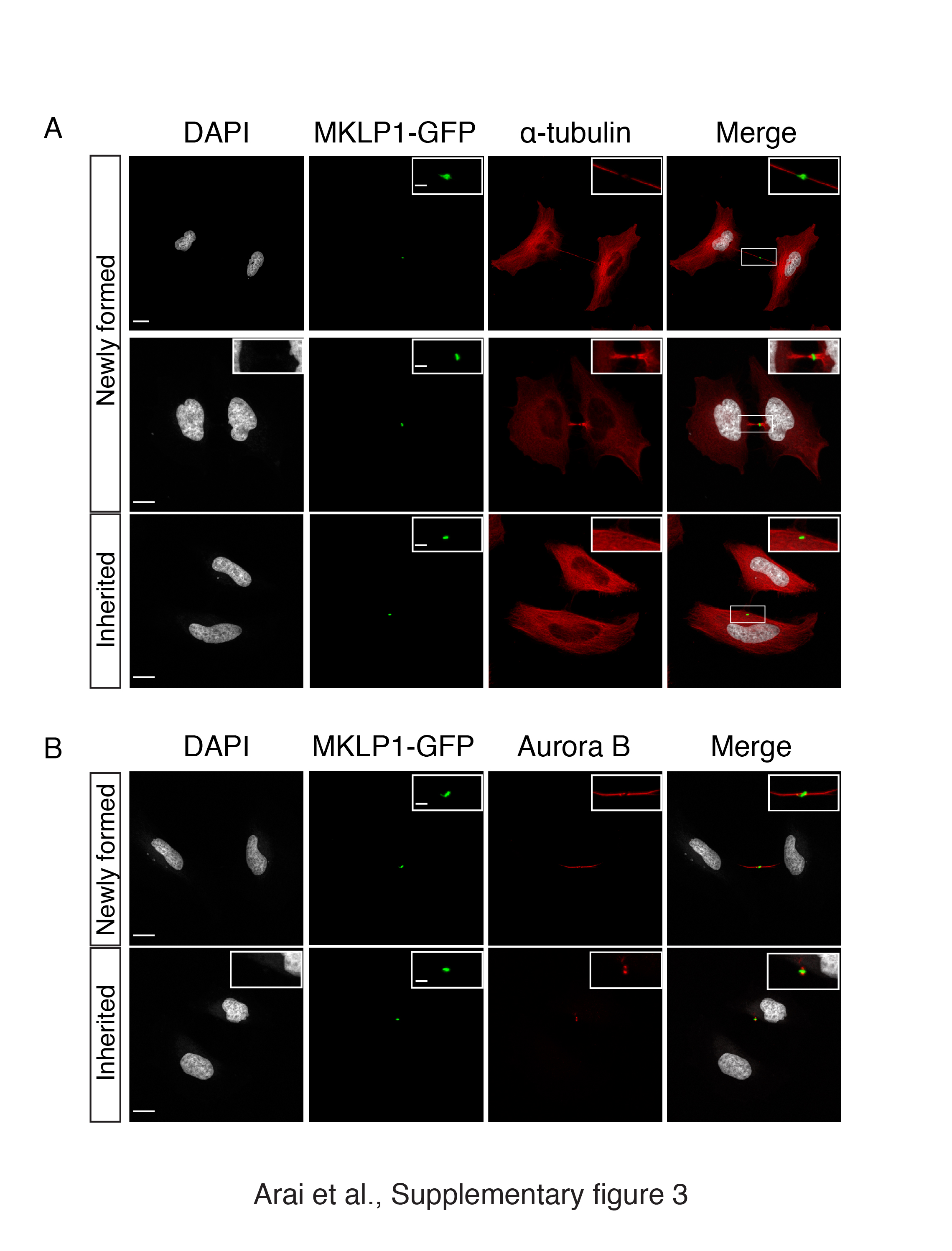

Supplement: Supplementary Figure 3 — Expression of midbody markers in MKLP1-GFP HeLa cells. (A) Fluorescence for MKLP1-GFP (green) and immunofluorescence for α-tubulin in telophase (2 upper panels) and interphase (lower panels) cells combined with DAPI staining (white). Scale bars, 10 μm. (B) Fluorescence for MKLP1-GFP (green) and immunofluorescence for Aurora B kinase in telophase (upper panels) and interphase (lower panels) cells having midbody combined with DAPI staining (white). Scale bars, 10 μm. Insets, magnifications of newly formed midbodies with telophase-bridge between the two daughter cells intact [upper panels in (A,B)] or inherited, monolateral midbodies with no apparent cytoplasmic bridge to the other daughter cell [lower panels in (A,B)]. Scale bars, 1 μm. [file Image3.JPEG]
